# Intracellular Follicle-Stimulating Hormone Receptor Trafficking and Signaling

**DOI:** 10.3389/fendo.2018.00653

**Published:** 2018-11-02

**Authors:** Niamh Sayers, Aylin C. Hanyaloglu

**Affiliations:** Department Surgery and Cancer, Institute of Reproductive and Developmental Biology, Imperial College London, London, United Kingdom

**Keywords:** GPCR, FSH receptor, endocytosis, signaling, trafficking, cAMP, endosome

## Abstract

Models of G protein-coupled receptor (GPCR) signaling have dramatically altered over the past two decades. Indeed, GPCRs such as the follicle-stimulating hormone receptor (FSHR) have contributed to these new emerging models. We now understand that receptor signaling is highly organized at a spatial level, whereby signaling not only occurs from the plasma membrane but distinct intracellular compartments. Recent studies in the role of membrane trafficking and spatial organization of GPCR signaling in regulating gonadotropin hormone receptor activity has identified novel intracellular compartments, which are tightly linked with receptor signaling and reciprocally regulated by the cellular trafficking machinery. Understanding the impact of these cell biological mechanisms to physiology and pathophysiology is emerging for certain GPCRs. However, for FSHR, the potential impact in both health and disease and the therapeutic possibilities of these newly identified systems is currently unknown, but offers the potential to reassess prior strategies, or unveil novel opportunities, in targeting this receptor.

## Introduction

The follicle-stimulating hormone receptor (FSHR) belongs to the superfamily of G protein-coupled receptors (GPCRs). With more than 800 members in humans they represent the largest family of signaling receptors and a major, successful, drug target (1). The canonical model of GPCR signaling is via plasma membrane localized receptors coupling to distinct heterotrimeric G proteins. However, we now understand the signaling pathways activated by GPCRs are much more complex to mediate the many distinct functions these receptors play in all physiological systems, but also equally important to decipher is how such signal pathways are regulated. These novel mechanisms are beginning to open up new avenues for therapeutic exploitation. One mechanism that not only contributes to the diversification of signaling but how cells decode or specify these signals is membrane trafficking. Classically, membrane trafficking was viewed as a mechanism to regulate sensitivity of a tissue to hormone, by altering the level of surface receptor either through ligand-mediated endocytosis in to the cell, and/or reduced biosynthetic trafficking of newly synthesized receptor. However, intracellular membrane compartments have been shown to represent additional signaling platforms for many kinds of receptors, including GPCRs such as FSHR.

This review will discuss our current understanding of the molecular mechanisms and signaling roles of membrane trafficking of FSHR, and how gonadotropin hormone receptors have shed light on novel cell biological pathways potentially applicable to many GPCRs. We will primarily focus on post-endocytic intracellular trafficking, and then discuss how this novel cell biology could shed light on specific facets of FSH/FSHR function and its implications to endocrine function.

## Classical regulation of FSHR signaling pathways

### Pleiotropic G protein signal profiles of FSHR

FSHR is a member of the glycoprotein hormone receptor subfamily of the rhodopsin-like, or Class-A, family of GPCRs, which comprise a unique subgroup within the Class A family due to their leucine-rich repeat-containing extracellular ectodomain. Furthermore, the high glycosylation status of its ligand FSH, like other glycoprotein hormones, makes them the most complex of protein hormones. Glycoprotein hormones are heterodimers that consist of a common α-subunit and a β-subunit that confers hormone specificity. These subunits are linked non-covalently and both subunits are subjected to N-glycosylation that can alter their bioactivity (2). For FSH, two naturally occurring glycoforms have been identified, the hypo-glycosylated FSH^21/18^ and the fully glycosylated FSH^24^ (3), which have distinct activities [recently reviewed in (4)] and may be of significance to the trafficking pathways and intracellular signaling to be discussed below.

**Figure 1:**
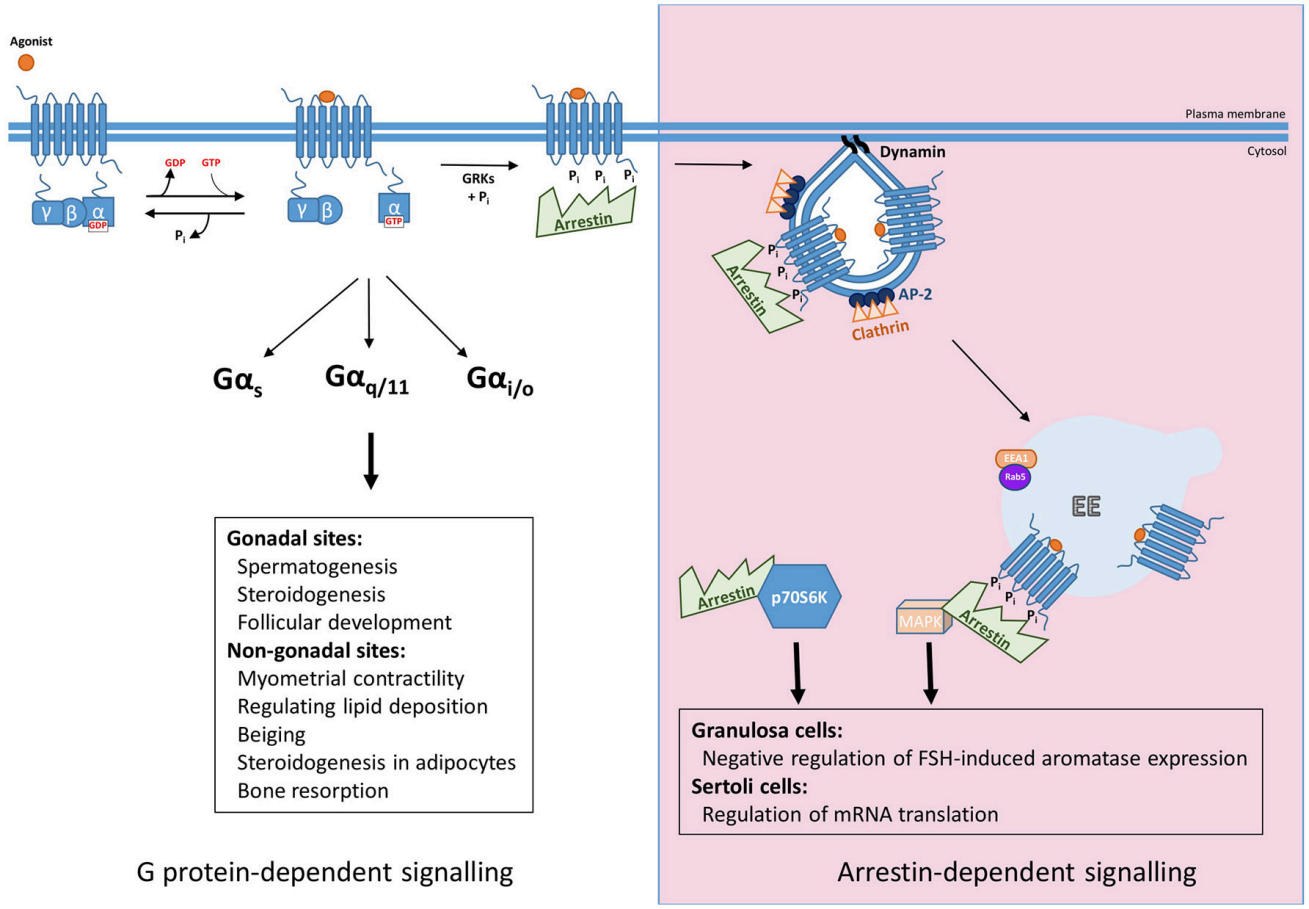
Summary of G protein and arrestin-mediated signal pathways activated by FSHR. Upon ligand binding FSHR has been reported to couple to Gαs, Gαq/11, and Gαi/o heterotrimeric G proteins to mediate its downstream effects, both those in the gonads such as spermatogenesis, steroidogenesis, and follicular development, and more recently at non-gonadal sites, see text for further details. The archetypal view of GPCR signaling occurs in a G protein-dependent manner, however, agonist-activated, phosphorylated receptor recruits arrestin, for G protein signal desensitization and internalization via clathrin-coated pits. Furthermore, arrestin mediates signaling pathways independent of G proteins. This can occur through the scaffolding protein binding signaling proteins such as components of MAPK pathways after G protein activation, or independent of FSHR by complexing and activating ribosomal protein, p70S6K.

FSHR plays critical roles in reproduction, identified via numerous studies in both animal models and disease causing mutations in humans reviewed in Huhtaniemi and Themmen(5) and Jonas (6), but also extragonadal functions in uterus, adipose and bone, have been identified. In the gonads, FSH binds its receptor in testicular Sertoli cells and ovarian granulosa cells, where they regulate follicular development, steroidogenesis and spermatogenesis. Additional roles of FSHR in non-gonadal sites include myometrial contractility, regulation of lipid deposition, beiging and steroidogenesis in adipocytes and bone resorption functions in osteoclasts (7–10). The primary G protein pathway classically associated with the gonadally expressed receptor is the Gα_s_/cAMP/PKA (11). However, FSHR has also been shown to couple to additional G protein pathways: Gα_q/11_, which leads to activation of phospholipase C, leading to production of diacylglycerol and inositol trisphosphate second messengers, the latter of which leads to increases in intracellular calcium levels. However, coupling of Gα_q/11_ to human FSHR is weaker than rodent FSHR and requires high receptor and hormone levels (12–14). Recently it was reported that in pregnant myometrium FSHR levels are coupled to Gαs to increase cAMP, a second messenger known to quiesce the myocytes, but during labor FSHR levels increase and the signaling switches to the pro-contractile calcium pathway, possibly by Gαq/11 coupling (7). Other G protein pathways can also be activated by FSHR. In Sertoli cells and osteoclasts FSHR has been reported to couple to Gαi/o family members, such as Gα_i2_, with subsequent MEK/Erk, NF-κB, Akt activation (9, 15, 16). Thus, like many GPCRs, FSHR exhibits the potential to activate G protein signaling in a pleiotropic manner, and such diversity in G protein signaling profiles may be tissue specific (Figure 1). The mechanisms underlying this tissue specificity in G protein coupling of FSHR may go beyond alterations in the levels of specific Gα subunits. Indeed, mechanisms such as GPCR homo and heteromerization and plasma membrane organization of receptors and signaling proteins such as in lipid rafts, are known to alter signal profiles of different GPCRs (17–19). FSHR is also subject to such mechanisms of signal diversity (20–22), although its role in directing tissue, or cell specific, responses is unknown.

### Arrestin-dependent desensitization, internalization and signaling

The mechanisms mediating regulation of GPCR/G protein signaling are critical in shaping, or programming, the G protein signal profile from the plasma membrane. Although models of this classic pathway of GPCR signal desensitization and internalization have rapidly evolved in the last 5 years, exhibiting increasing complexity, particularly as application of structural and super-resolution imaging are increasingly applied, the core features of this model (Figure 1) are still critical. Signal activation of GPCRs at the plasma membrane is regulated initially via a process of rapid desensitization, followed by internalization via clathrin-coated pits (CCPs). The initial step in this model involves receptor phosphorylation on serine/threonine residues by second messenger-activated kinases and/or GPCR kinases (GRKs). It is both the activated and phosphorylated receptor that enables recruitment of the adaptor protein arrestin from the cytoplasm. Arrestins are a family of adaptor proteins with an increasing array of functions, both for GPCRs and non-GPCR mediated signaling. The family contains four isoforms, where arrestin-1 and−4 are restricted to the visual system and are also termed “visual arrestins” (23, 24), whereas the other two isoforms, arrestin-2 and -3, also called β-arrestin-1 and -2, are ubiquitously distributed and bind many GPCRs (25, 26). The arrestin-bound receptor desensitizes signaling via the uncoupling of receptor from its cognate G protein. Rapid arrestin-dependent internalization occurs by firstly inducing receptor clustering into CCPs, via its ability to bind receptor, clathrin heavy chain and core clathrin adaptor proteins (namely the β2 subunit of AP2) (27). This model, comprehensively described across many reviews, have discussed ways in which GPCRs may employ this system in distinct manners, such as differential phosphorylation of intracellular GPCR domains, primarily at the carboxy terminal tail (C-tail), alters association kinetics of GPCR to arrestin and its subsequent impact on receptor activity (28, 29). However, more recent structural and functional studies demonstrating the distinct modalities that arrestin can complex with GPCR and the G protein bound GPCR (30–34) have unveiled novel features of regulation that could be highly pertinent for FSHR, and will be discussed below.

FSHR signaling is regulated by this canonical GRK/arrestin model. As discussed above, internalization and desensitization are mediated by GRK phosphorylation, followed by arrestin binding. This is true for FSHR, where a cluster of five serine/threonine residues in its C-tail were identified as the key sites for GRK 2, 5, and 6 phosphorylation (35). Interestingly, while GRK 2 is predominantly involved in FSHR arrestin-mediated desensitization, GRKs 5 and 6 also promote arrestin binding but for scaffolding signaling proteins and signal activation (36). For FSHR, specific threonine residues in the third intracellular loop have been identified to dictate their rate of internalization and arrestin sensitivity, or binding (37). Further, there are differences in how FSHR engages with the GRK/arrestin mechanism between rodent and human receptors and also when compared to its “sister receptor” the LH receptor (LHR) that has unveiled specific structural motifs in FSHR involved in its internalization rate. The rodent FSHR internalizes faster than the rodent LHR and the human FSHR. Creating chimeras between receptors revealed that six amino-acids in transmembrane 4, intracellular loop 3 and transmembrane 7 determine internalization of rodent FSHR to be 3 times faster than human FSHR, and its sensitivity to arrestins in enhancing receptor internalization (38). In addition, a serine/threonine phosphorylation cluster in the rodent FSHR C-tail is also involved in arrestin binding and arrestin-dependent internalization and desensitization, but not MAPK signaling (36). The importance of the third intracellular loop and C-tail residues in arrestin binding is interesting especially given recent key developments in our molecular understanding of how arrestin engages with GPCRs to mediate its distinct functions. Structural studies have now shown that arrestin engages active receptor in at least three forms, via the phosphorylated C-tail, the receptor core, or both. These forms have helped to explain how arrestin can mediate G protein uncoupling, internalization and yet also facilitate signaling when either as a stable or transient GPCR/arrestin complex (30, 31, 33, 39). These differences in arrestin binding to receptor could suggest that for FSHR core and C-tail interactions with arrestin may mediate its functions in an opposing manner than described for other GPCRs. However, as will be covered in the next section, arrestins also have roles in driving gonadotropin hormone receptor signaling and there may be alternate FSHR/arrestin conformations to mediate its various functions.

The role of arrestins as scaffolding proteins for different signaling proteins is now well recognized. The primary signaling pathway studied that is activated by such arrestin scaffolds is the MAPK pathway (40–42). For FSHR, ligand-dependent ERK signaling exhibits a sustained profile whereby the early activation is dependent on Gαs/PKA activation while the sustained response requires arrestins [(36) and Figure 1]. Likewise, the ligand-dependent interactions between FSHR and arrestin are also sustained as measured by BRET (43), although there have been no image-based data confirming sustained, or internalized FSHR/arrestin complexes in cells. Given arrestins are involved in FSHR internalization, its role in signaling strongly suggests additional scaffolding roles of this adaptor protein, and/or a requirement of receptor internalization of ERK signaling as has been demonstrated for LHR (44). However, a partial inhibition of FSHR internalization via arrestin and dynamin dominant negative mutants did not impact ERK signaling (45). Although more work is required to clarify how arrestins mediate G protein-independent FSH signaling, perhaps recent studies could provide clues to a mechanistic understanding of how FSHR mediates sustained arrestin-dependent MAPK responses. The β1-adrenergic receptor (β1AR) is a GPCR that induces a sustained ERK profile mediated by arrestins, but only transiently associates with arrestin at the plasma membrane, while arrestin remains associated within CCPs in order to activate signaling in a sustained manner (46). The authors demonstrated that arrestin transiently binds to the core of β1AR only, which induces a conformational change in arrestin resulting in its capture by binding phosphoinositides in the plasma membrane and clathrin associated adaptor proteins (33). Given the sustained ERK signaling profile of FSHR, that the third intracellular loop residues are involved in arrestin binding, while C-tail sites do not regulate ERK signaling (37, 47, 48) and further similarities in these receptors with the β1AR in their post-endocytic pathways [see section Post-endocytic sorting and endosomal signaling of FSHR from a novel compartment; the very early endosome (VEE)], it is possible that arrestin engages with FSHR in a similar manner. Alternatively, or in addition to this receptor-independent arrestin signaling complex, is the recent elegant report demonstrating that FSHR activates p70S6K within an arrestin complex constitutively assembled with a p70S6K/ribosomal protein S6 (rpS6) to regulate mRNA translation (49). This study supports recent structural findings that arrestin, after its dissociation from receptor, can maintain various active conformations (50), but also suggests that receptor-independent “active” arrestin complexes may not only be a feature of CCPs but also other subcellular locations.

The ability of GPCRs to activate more than one pathway of signaling, such as G protein and arrestin-mediated signaling via the stabilization of a certain active conformation of the receptor, is termed signaling bias (28) and is of high pharmacological interest. Bias can occur through different ligands (ligand bias) or even receptor mutations (receptor bias) (51). From a therapeutic perspective, the ability to specifically target the desired cellular effects, through one pathway, without activating unwanted side effects has been shown to be a feasible strategy for certain GPCRs. In terms of G protein vs. arrestin-mediated signaling, recent studies have challenged this model for certain GPCRs, suggesting that G protein activation is still an essential upstream event of arrestin-dependent ERK signaling (52, 53). Perhaps an argument against a requirement for G protein activation in arrestin-mediated signaling for FSHR is via the observation that lowering expression levels of the receptor to a level where there is no detectable cAMP signaling can induce bias to arrestin-dependent signaling (54). This was first revealed by studies on the A189V FSHR mutant, which is expressed at very low levels on the cell surface and is non-functional with respects to Gαs/cAMP signaling (55). Yet, when both A189V mutant and wild-type FSHR are expressed at equivalent low levels they are only able to trigger G-protein independent MAPK activation (54).

For biased signaling to be therapeutically explored for FSHR, there must be a well characterized understanding of the *in vivo* role of arrestin in FSHR signaling. So far, it has been demonstrated that in Sertoli cells arrestin may regulate mRNA translation and a possible negative regulation of FSH-induced aromatase expression in rodent granulosa cells (via manipulation of GRK6 levels as an upstream step in arrestin binding) (56) (Figure 1). This latter study is perhaps corroborated by findings in an immortalized human granulosa tumor cell line, whereby arrestins negatively regulate Gαs/cAMP/PKA pathway, not in terms of classical desensitization, as in these cells gonadotropin-mediated ERK signaling via arrestins was evident in the absence of cAMP signaling. Specifically, FSH, but not LH, dependent apoptosis occurred by cellular depletion of arrestins, due to increases in cAMP/PKA signaling, thus suggesting a role for arrestins in regulating balance between cell proliferation and apoptosis (57). While promising, further work needs to be conducted to evaluate the potential benefit of arrestin-based biased agonism.

### Post-endocytic intracellular trafficking pathways of FSHR

Following internalization, GPCRs are trafficked to endosomes where they are sorted to either a plasma membrane recycling pathway, or to the lysosomal pathway for degradation. Such pathways program the temporal profile of G protein signaling, by regulating resensitization/hormone recovery (recycling) or permanent signal termination (degradation), Additionally, the sorting fate of a GPCR pharmacologically manipulated and altered in disease (58). However, we now know that the endocytic system does not only regulate the surface density of receptors but that these divergent, and complex, sorting pathways have direct roles as platforms for signaling, including G protein signaling (59–61). This will be further discussed in section Postendocytic sorting and endosomal signaling of FSHR from a novel compartment; the very early endosome (VEE). The mechanisms that underlie these divergent post-endocytic fates are tightly regulated at multiple levels and are interlinked with the receptors own signaling. These complex mechanisms have recently been reviewed by us and others (61–64) and will not be described in detail here except to illustrate core features that enable discussion of current understanding of FSHR sorting and intracellular signaling.

The textbook model of cargo sorting depicts the Rab5 early endosome (EE) as the common post-endocytic compartment from which receptors are first sorted to opposing fates. GPCRs sorted to a degradative pathway following internalization are trafficked from EEs to Rab7 positive late endosomes. Receptors are involuted into vesicles within the lumen of these endosomes, to form multivesicular bodies (MVBs). MVBs will then fuse with lysosomes resulting in protein degradation. GPCRs will engage with this pathway with distinct kinetics and for those receptors targeted to the recycling pathway, chronic ligand stimulation will reroute receptors to this degradative pathway as part of the mechanism of downregulation. Classically, lysosomal targeting of different receptors is via ubiquitination at lysine residues and engagement with endosomal sorting complex required for transport (ESCRT)-dependent degradation, however, GPCRs exhibit ubiquitin-independent and ESCRT-independence in their mechanisms of degradation [reviewed in (65, 66)]. GPCRs targeted to a rapid recycling pathway are sorted from EEs to Rab4 positive recycling endosomes. An important feature of GPCRs targeted to recycling pathways, is that this is regulated by interactions with specific sequences in the GPCR C-tails, also termed sequence-directed, or regulated, recycling. This mode of recycling is distinct from recycling of other kinds of membrane cargo, e.g., transferrin receptor that does not require its C-tail for recycling and occurs with the bulk membrane flow (default recycling). The distal C-tail receptor sequences are not only essential for recycling, but if fused to the carboxy-terminus of a GPCR sorted to a degradative pathway, it will reroute that GPCR to the recycling pathway. There are no common sequences that determine whether any GPCR undergoes regulated recycling, as they are highly divergent. However, several recycling sequences identified, such as first identified with the β2-adrenergic receptor (β2AR), correspond to a type 1 (PSD95)/discs large (Dlg)/zonula occludens-1 (Zo-1) (PDZ) binding sequence or “PDZ ligand,” specifically S/T–X–Φ, (where Φ is any hydrophobic residue) (67, 68). PDZ proteins are scaffold proteins and for GPCRs that bind PDZ proteins, they are often able to bind more than 1 PDZ protein (69), suggesting these sequences and interactions may have additional functions to directing receptors to the recycling pathway. For the β2AR the interacting PDZ-domain containing protein partner responsible for recycling is the endosomally localized PDZ protein, sorting nexin-27 (SNX27) (70). As mentioned above, these recycling sequences are very distinct amongst receptors, so there are several examples of GPCRs targeted to a recycling pathway that do not contain PDZ type 1 ligands or any other recognizable motif, and hence for many their corresponding interacting protein partners are unknown (58, 71). This is also the case for the FSHR whereby both rodent and human FSHR are recycled back to the plasma membrane via specific C-tail sequences that does not indicate any potential binding partners such as a PDZ protein (72). More recently a role for palmitoylation in FSHR sorting has been proposed (73). In FSHR it is known that there are 2 conserved and 1 non-conserved cysteine in the FSHR C-tail that were all palmitoylated, but only 1 of the conserved cysteines (cysteine 629) affected receptor function by impairing cell surface expression (74). A follow up study demonstrated that mutation of all three cysteines to glycine significantly impaired biosynthetic trafficking of the receptor to the plasma membrane and thus exhibited reduced signaling. Interestingly, the receptor that was transported to the plasma membrane exhibited similar internalization kinetics but impaired recycling, the receptor thus being routed to the degradative pathway (73). This suggests that altering palmitoylation of the receptor changes the ability of the C-tail, and presumably the distal recycling sequence, to interact with key machinery that mediates its sorting, but also indicates that a cell could alter FSHR trafficking, and subsequently signaling responsiveness, through alterations in these post-translation modifications.

While the above describes that the recycling pathway of FSHR, and other GPCRs, involves a one-step mechanism with its recycling sequence and interacting partner, which may be unique to a given GPCR, we now know that both common and receptor-specific post-endocytic mechanisms exist. Furthermore, sequence-directed recycling (and lysosomal sorting) occur via a complex, multi-step system, with the GPCR's own signaling playing a key role in driving receptors to these distinct cellular fates, in addition to trafficking regulating the signal from that receptor. This interconnected property of post-endocytic trafficking and GPCR signaling has been highlighted recently through studies of the gonadotropin hormone receptors and will be discussed next.

## Post-endocytic sorting and endosomal signaling of FSHR from a novel compartment; the very early endosome (VEE)

The internalization of GPCRs into the endocytic network is no longer viewed as a mechanism to only control plasma membrane signaling but to also provide additional platforms to continue or reactivate signaling from intracellular compartments, including G protein signaling. Furthermore, this spatial control of signaling has been shown to be important for cells to decode common second messenger signaling molecules, such as cAMP signaling, activated by many GPCRs, into specific downstream cellular functions (59–61). Intracellular signaling is also important for the FSHR, however, it is through studies on human LHR has unveiled how important, and tightly regulated, the compartmentalization of receptors within the complex endomembrane network is to receptor endosomal signaling.

### Discovery of the VEE; a tale of serendipity

**Figure 2:**
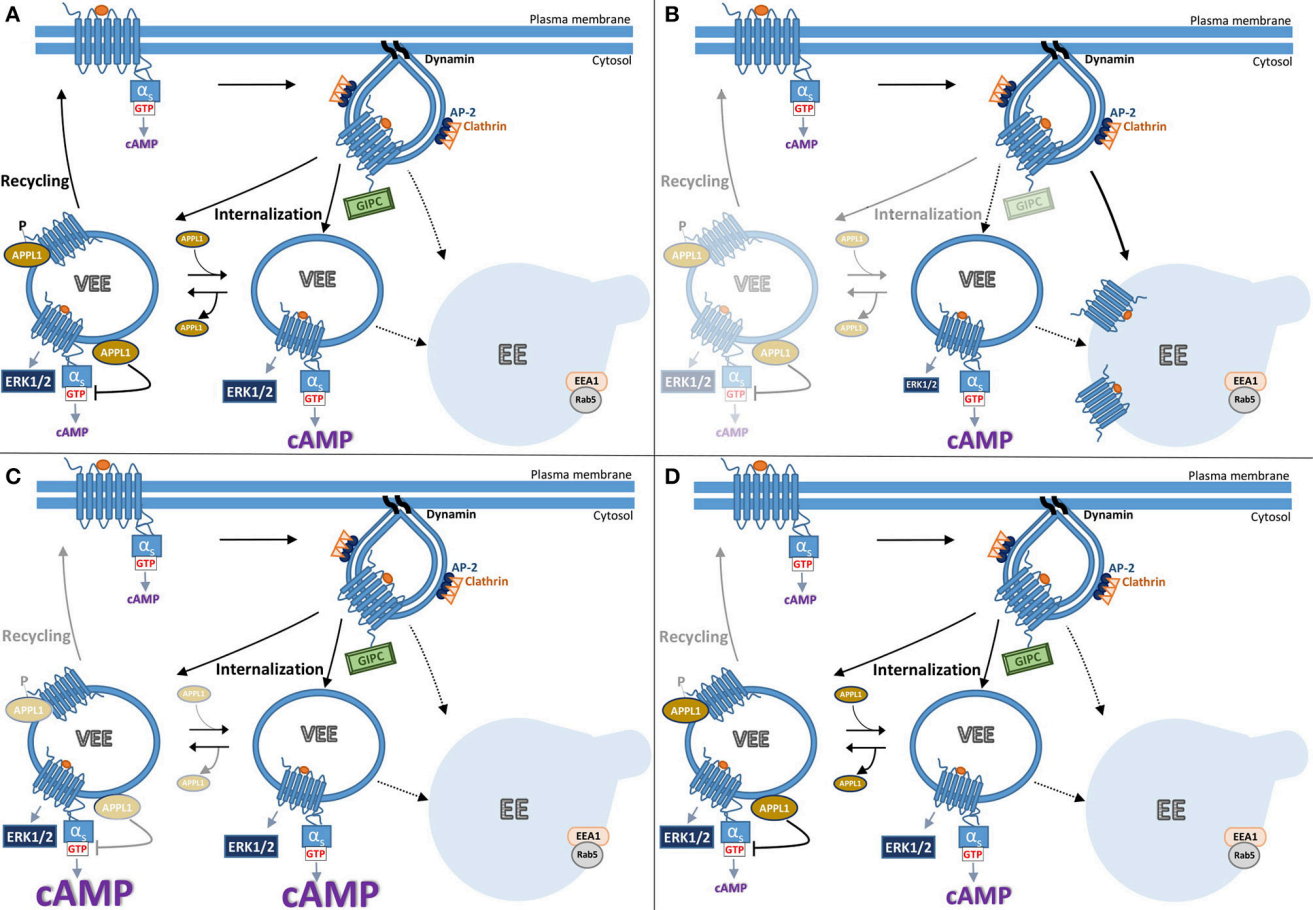
Model summarizing current understanding of gonadotropin hormone receptor post-endocytic pathways and receptor signaling from VEEs. The trafficking of FSHR and LHR to the VEE is inextricably linked to the receptor's signal output, whereby the manipulation of which at distinct steps results in different trafficking and signal profiles. **(A)** Following ligand-activation FSHR internalizes to the very early endosome (VEE). The VEE differs from the early endosome (EE) in its smaller size and neither contain EEA1 nor Rab5, classical markers for the EE. During receptor-mediated endocytosis of FSHR into a clathrin-coated pit, the PDZ domain protein, GIPC, is recruited at the cytosolic interface of the GPCR. Receptor then enters the complex endosomal network where it is primarily localized to the VEE. There are two types of VEE depicted, one contains the adaptor protein, APPL1 (see text), and one is without. GIPC dissociates before FSHR enters the VEE. From the VEE receptor is able to elicit downstream signaling cascades, including cAMP generation and ERK1/2 activation. This cAMP/PKA signal phosphorylates APPL1 on Serine 410. Receptor is trafficked to APPL1 positive VEEs, where the unphosphorylated APPL1 negatively regulates endosomal cAMP signaling. The phosphorylated APPL1 is required for receptor recycling back to the plasma membrane. **(B)** The receptor can be rerouted from the VEE to the EE by the loss of GIPC or disruptions in the receptors ability to interact with this PDZ protein. Loss of GIPC results in the trafficking of the receptor from the VEE pathway to the EE and loss of plasma membrane recycling. While endosomal cAMP signaling is not affected, ERK signaling profile is more transient as the receptor only rapidly passes through the VEE to the EE. **(C)** Loss of APPL1 does not alter the endosomal organization of the receptor but inhibits recycling. The “trapped” receptor in the VEE compartment results in increases in endosomal cAMP signaling due to APPL1's role in negative regulation of G protein signaling, but without impacting ERK signal profile. **(D)** Manipulating the ability of APPL1 to be phosphorylated on Serine 410, either by inhibition of PKA activity or mutation of serine 410 to alanine, specifically inhibits recycling but not cAMP endosomal signaling.

Many GPCRs, including the human FSHR and LHR undergo the regulated recycling pathway described in Section Post-endocytic intracellular trafficking pathways of FSHR. Our studies first identifying the VEE with the gonadotropin hormone receptors were initially driven as way to understand why GPCRs have distinct recycling sequences if there is a common primary function, i.e., sorting to the recycling pathway. Thus, comparisons were first made between the β2AR and LHR that initially seem quite similar in their signaling and trafficking profiles (44). Both are Gαs-coupled receptors that internalize via arrestin/clathrin pathways and undergo sequence-directed recycling. However, they have distinct C-tail recycling sequences and bind different PDZ proteins for their sorting, SNX27 for β2AR and GIPC (Gαi-interacting protein, C-terminus) for LHR (68, 70). Unexpectedly, when agonist-induced LHR endocytosis was monitored by live confocal microscopy imaging it was evident this receptor trafficked to endosomes closer to the plasma membrane that were approximately a third of the diameter of endosomes containing β2AR. FSHR and the β1AR also internalized to these small endosomes. The identity of this compartment, in terms of the proteins or adaptors that traffic there are poorly understood, except they are distinct from those classically found in the EE and EE intermediates such as EE antigen 1, phosphatidylinositol-3 phosphate (a lipid enriched in the EE membrane) and Rab5 (44). As it was previously established that LHR sorting to the recycling pathway required its C-tail interaction with the PDZ protein GIPC, the hypothesis was that this protein interaction also directed LHR to these small endosomes. Indeed, truncation of the LHR recycling sequence or knockdown of GIPC levels inhibited recycling and also rerouted receptor to the larger EEs (Figures 2A,B). As interactions with GIPC occurred only early on during receptor clustering in to CCPs, further supported a model whereby LHR recycling must occur from these small endosomes, we then termed very early endosomes (VEEs) (44). While the discovery of a new cellular compartment was very unexpected, our original aim of understanding the role of these different GPCR recycling sequences was also in part addressed, as it highlights these sorting sequences may encode functions at distinct steps in the endocytic trafficking of a GPCR, and not only sorting from endosomes. In this case, it was sorting to distinct populations of endosomes. It is well known that trafficking regulates signaling, thus what were the signaling functions of the VEE? Surprisingly, when LHR trafficking was rerouted away from VEEs to EEs, via inhibiting interaction with GIPC, the ligand-induced cAMP signaling was not affected but ERK signaling profile was altered from a sustained to a transient one (Figure 2B) (44). Given that both acute and sustained ERK signaling was dependent on internalization, suggests that under conditions when GIPC is depleted, the receptor is rapidly routed through the VEE, but not maintained in this compartment due to its trafficking to EEs, hence the altered temporal ERK signaling profile (see Figure 2B). It also highlights the interconnectivity of endomembrane systems; indeed, we also demonstrated in this study that the transferrin receptor can internalize through the VEE on its way to the EE. Intriguingly, the ligand-induced ERK signaling of β1AR and FSHR was also affected by GIPC knockdown. While β1AR is known to interact with GIPC via its C-tail (75), this result was unexpected for FSHR since its recycling sequence contains no PDZ ligand and there are no prior reports of its interaction with GIPC. However, FSHR is known to directly interact with the adaptor protein containing PH domain, PTB domain, and leucine zipper motif (APPL1) (12, 76, 77), and so far, APPL1 is the only protein identified that localizes to a subpopulation of VEEs. Given APPL1 can directly bind GIPC (78) this may underlie the GIPC-dependent nature of ERK signaling by FSHR.

### The role of APPL1 in FSHR and VEE function

To date, APPL1 is the only known protein present on the VEEs, as the PDZ protein GIPC only associates during the very early steps of endocytosis at the CCP (44). APPL1 is a well-studied adaptor protein comprised by multiple protein and membrane interacting domains (79). Prior studies have shown it localize to EE Rab5 compartment but at an intermediate step prior to conversion of endosomes to EEA1 positive endosomes (80). APPL1 is reported to localize to other compartments including vesicles that do not have Rab5 (81), akin to what we have observed with the gonadotropin hormone receptors and the VEE (44). APPL1 displays multiple, and integrative functions in cargo trafficking and receptor signaling, as evident from the numerous reported interactions including Rabs, receptors such as FSHR, EGF receptor, insulin receptor, adiponectin receptor, androgen receptor, kinases and phosphatases, like protein kinase B and PtdIns(3)P kinase, and PDZ proteins like GIPC (82). Prior to our reports on the VEE, APPL1 was shown to form a complex with FSHR, not via the C-tail but via three specific residues in its first intracellular loop (76). In addition, this complex contained additional adaptor and signaling proteins, including APPL2, Akt, and FOXO1. Such a complex was shown to propagate FSH-induced PI3K/Akt signaling, IP_3_ production and calcium release (12, 76, 77, 83). This is consistent with APPL1's roles in positive regulation of signaling (78). These signal pathways in FSHR/APPL1 complexes remain to be studied in the context of the VEE where focus so far has been primarily on cAMP and MAPK.

APPL1 plays critical roles in gonadotropin receptor trafficking and endosomal cAMP signaling from the VEE (84). As described above, interactions with GIPC are essential for directing receptors to the VEE (Figure 2B). In contrast, APPL1 is not required for GPCR localization to the VEE but is essential for LHR and FSHR recycling (Figure 2C). Interestingly, it is the receptor's own cAMP/PKA signaling that drives this APPL1-dependent recycling (60). The mechanism underlying this requirement is that LH-mediated PKA activation must phosphorylate APPL1 at serine 410 for the receptor to recycle back to the plasma membrane. This raised the possibility that LHR activates signaling from the VEE as it would provide a means for high localized regulation of APPL1 (phosphorylation) at the endomembrane, and perhaps specific populations of APPL1. Inhibiting internalization of LHR almost completely abolished ligand induced increases in cAMP, a finding corroborated using a nanobody biosensor that recognizes active Gαs (85), which localized to a subpopulation of LHR endosomes (84). If the primary location of cAMP signaling from these receptors was the VEE, then inhibition of recycling should enhance cAMP signals. Indeed, this is the case when cellular levels of APPL1 are depleted for all known VEE-targeted receptors (LHR, FSHR, and β1AR, see Figure 2C). However, ERK signaling (strength and kinetics) is not affected under these conditions and inhibition of LHR recycling via a PKA inhibitor (as APPL1 phosphorylation is needed for recycling) has no effect on LHR-cAMP signaling. Thus, APPL1 has an additional role in negatively regulating cAMP from VEE targeted GPCRs (84). How APPL1 controls GPCR/Gαs coupling is still unknown, yet intriguingly it is the unphosphorylated form of APPL1 that mediates this, indicating that distinct populations of APPL1, phosphorylated and unphosphorylated forms, have opposing functions on VEE-targeted receptors (Figure 2). Furthermore, it highlights that endosomal signaling must be “switched off” prior to receptor recycling, as has been shown in GPCR endosomal signaling from the EE and the role of the retromer complex (86, 87). Overall, the VEE-network displays exquisite control of signaling and trafficking via the actions of APPL1. There are many outstanding molecular questions for this system, including if there are any roles for the related protein APPL2, which has both common and distinct functions to APPL1, especially as FSHR forms a complex with both adaptor proteins (77). Are other signal pathways that FSHR is known to activate, such as Gαi signaling (see section Classical regulation of FSHR signaling pathways), also regulated at the level of the VEE and by APPL1? Another question these studies have raised is why is such complexity within the endosomal system required? One possibility is that it enables a cell to alter receptor activity at many levels and potentially in a pathway specific manner (location bias in signaling), perhaps in response to physiologically relevant changes in its extracellular environment (e.g., dynamic hormonal environment during menstrual cycle) to pathological changes in disease. This is illustrated in Figure 2, whereby altering levels of key adaptor proteins GIPC and APPL1, alters receptor signaling to ERK or cAMP respectively (Figures 2B,C), or at the level of APPL1 regulation of PKA mediated phosphorylation, would result in a distinct trafficking and signal phenotype (Figure 2D).

Overall, compartmentalization of internalized gonadotropin hormone receptors, and indeed many other GPCRs, mediates their signal activity at specific intracellular sites, and represents a mechanism for cells to diversify signaling even from the same pathway (e.g., cAMP/PKA) to possibly distinct functional consequences. It also raises the intriguing possibility that GPCR activity, can be reprogrammed by the cell to alter the compartment the receptor is targeted to via altering expression of key proteins in this pathway, e.g., GIPC, APPL1 (Figure 2). Such a model may explain how FSHR activity in the gonads, commonly mediated by cAMP/PKA, underlies distinct functions but also specificity of this pleiotropically coupled GPCR.

## Outstanding questions and future perspectives

These recent advances in FSHR function from its distinct roles in extragonadal tissues and novel cell biological functions demonstrates that FSHR is a good example of a GPCR that achieves signal diversification via multiple strategies and a prototype receptor for understanding novel facets of GPCR function. Whether such pathways are then perturbed in disease and if we can harness these properties of GPCRs therapeutically needs to be addressed.

While much has been uncovered about novel intracellular trafficking systems, there are numerous outstanding questions at the molecular level. A major one to be tackled in the future is the identifying the molecular composition (both protein and lipid content of the membrane) of the VEEs, as so far APPL1 is the only known protein to reside there and is only present in ~50% of the VEE population (84). While not a trivial task to unpick, recent developments in proteomics such as the application of engineered ascorbic acid peroxidase (APEX)-mediated approaches that can capture the local protein network of a receptor with high spatial-temporal resolution as it traffics through the endocytic system (88), may be able to uncover specific VEE proteins and whether such proteins are core to these endosomes, or receptor-specific. A critical outstanding question is understanding the downstream role of VEE targeting, and could there be any clues from disease-causing mutations in the gonadotropin hormone receptors? It is likely that the VEE and APPL1-dependent regulation is a conserved mechanism across cells, as for many membrane trafficking pathways. In primary human endometrial stromal cells, LHR could traffic to VEEs and recycle in an APPL1-dependent manner (84), although the downstream functional role/s in the endometrium, a tissue reported to express both functional LHR and FSHR are unknown. A role for APPL1 in regulating gonadotropin action has been reported in the ovary whereby knockdown of APPL1 in bovine theca cells enhances LH-mediated androgen production (89). Given loss of APPL1 increases ligand-dependent cAMP signaling in HEK 293 cells (84) (Figure 2C), is consistent with this finding and could suggest that deregulated signaling by VEE/APPL1 has key physiological/pathophysiological roles in the ovary such as steroidogenesis and conditions where there is enhanced LH activity e.g., PCOS. Indeed, a direct role for gonadotropin hormone receptor endocytosis in LH-mediated cAMP signaling in the mouse ovarian follicle and the resumption of meiosis in the oocyte has been demonstrated (90). Specific roles for FSH/FSHR function will need to be investigated, given there are known disease causing mutations that alter FSHR signal desensitization and inhibit internalization (91). The reassessment of known mutations in the context of the VEE would be highly informative as tools for further understanding how FSHR engages with these intracellular trafficking and signaling mechanisms. However, such disease-causing mutations are rare, and from a translational perspective there would be value in assessing known single nucleotide polymorphisms that impact FSHR activity for potential alterations in VEE function. For example, the FSHR asparagine/serine polymorphism at position 680 (N680S), whereby in the general population approximately 60% are N680 and 40% S680, while in infertile individuals this is more 50/50. This SNP determines poorer responsiveness to FSH in women bearing the N variant compared to the S carriers that has been linked to temporal alterations in cAMP, ERK1/2 and CREB responses in human granulosa cells, whereby interestingly it is the N variant that exhibits faster signal properties (92, 93). These altered signal properties could result in altered arrestin-mediated signaling and/or altered sorting of FSHR to endosomal compartments. Although any potential alterations in intracellular trafficking at the level of the VEE are unknown, given the C-tail location of the SNP, located upstream of the distal recycling sequence of FSHR (72), one could predict that this variant may modulate interactions with protein partners that bind this distal recycling sequence, resulting in distinct post-endocytic fates and altered endosomal compartmentalization. The faster kinetics in signaling exhibited by the N variant may indicate for example, a lack of APPL1-dependent regulation in signaling and/or inability of GIPC to direct the receptors to the VEE. Given the more widespread functions of FSH that have been reported, this SNP may be predictive of other conditions, such as pre-term birth whereby carriers homozygous for the N variant, as evaluated from placental samples, had a significantly higher risk of pre-term birth than the S variant (94). This variant is not only of significance to female health but also to males. Infertile men carrying the 680N variant compared to the 680S respond better to FSH treatment as assessed by improved DNA fragmentation index of their spermatozoa (95). Furthermore, the increasing reports of FSHR in endothelial cells, bone and adipose (8, 9) may unveil future roles for altered spatial control of FSHR signaling in cancer, obesity, type 2 diabetes and osteoporosis.

The ability to pharmacologically exploit these new GPCR signaling models and target the intracellular signaling receptor specifically has been recently demonstrated for three distinct GPCRs. Employing cholestenol-conjugated antagonists, which accumulate in the lumen of endosomal compartments, can block endosomal signaling from the neurokinin 1 receptor and calcitonin-gene related peptide receptor and has been shown to be an effective nociceptive target in animal models (96, 97). Whilst a similar strategy to inhibit endosomal signaling of the protease-activated receptor 2 prevented hyperexcitability of pain receptors in the colon and thus has been proposed to be of therapeutic value in pain management of irritable bowel syndrome (98). These studies set an important precedent for the likely success of targeting intracellular signaling of GPCRs, however, we know GPCRs including FSHR exhibit pleiotropic signaling and the ability to also target intracellular signaling more specifically, either at a pathway level or endosomal compartment level (e.g., VEE vs. EE) could be valuable. In other words, to create biased intracellularly targeted compounds may offer avenues for more efficacious compounds with less side effects. For FSHR there may already exist avenues to develop such ligands, as a number of small molecule, orally available, and cell permeable, compounds have been produced (99), although their role in altering intracellular signaling of FSHR remain to be determined. It may not always be necessary to target intracellular receptors, and perhaps altering the endosomal fate of a receptor by targeting the plasma membrane receptor prior to its internalization may be advantageous, to also induce location bias. This could even be achieved through native ligands; for FSHR there are known glycovariants of FSH that exhibit distinct activities (4).

In summary, our understanding of the complexity of GPCR signaling pathways via the tight control of their intracellular location has been advanced through studies on the gonadotropin hormone receptors. Such mechanisms have highlighted the interconnected nature of these intracellular systems, and thus a primary future goal is to further understand the significance of these molecular systems to health and disease if they are to be of therapeutic value. The critical nature of intracellular sorting of FSHR to signaling has been demonstrated, so it is not a question of is it important, but rather how the intricacies of modulating receptor from one intracellular compartment to another impact specific functions *in vivo*. This would provide the opportunity to be able to target these intracellular signaling modalities with high precision, in order to create the next generation of therapeutics for reproductive medicine.

## Author contributions

All authors listed have made a substantial, direct and intellectual contribution to the work, and approved it for publication.

### Conflict of interest statement

The authors declare that the research was conducted in the absence of any commercial or financial relationships that could be construed as a potential conflict of interest.

## Abbreviations

APPL1: adaptor protein containing PH domain
PTB domain: and Leucine zipper motif
β1AR, β2AR: beta adrenergic receptor 1 or 2
CCP: clathrin coated pit
EE: early endosome
EGF: epidermal growth factor
EGFR: EGF receptor
ESCRT: endosomal sorting complex required for transport
GIPC: Gαi-interacting protein C terminus
GPCR: G protein-coupled receptor
LHR: luteinizing hormone receptor
PDZ: postsynaptic density 95/disc large/zonula occludens-1
PKA: protein kinase A
VEE: very early endosome.
